# 

**Published:** 1911-03-11

**Authors:** 


### CONTENTS.

| | PAGE |
|---|---|
| **Nerve in Surgery** ... ... ... ... ... ... ... ... | 673 |
| **Experimental Biology** ... ... ... ... ... ... ... | 674 |
| **Annotations—** | |
| The Smallpox Outbreak—The Notification of Leprosy—Surgical "Negligence" in France ... ... ... ... ... ... | 675 |
| **Medical Opinion and Movement** ... ... ... ... ... | 676 |
| **Hospital Clinics—** | |
| The Action of Certain Drugs on the Heart. By J. M. Fortescue-Brickdale, M.A., M.D., Oxon. ... ... ... ... ... | 679 |
| **Surgery—** | |
| A New Method of Colostomy ... ... ... ... ... ... | 682 |
| **General Practitioner's Column—** | |
| Benzoic Acid and the Benzoates as Urinary Antiseptics ... | 683 |
| Comedo Extractor ... ... ... ... ... ... ... | 684 |
| **"The Hospital" Medico-Sociological Supplement. No. III.—** | |
| The Reaction of Proposed Social Reforms upon our Hospital Systems .. ... ... ... ... ... ... ... ... | 635 |
| **News and Events of the Week** ... ... ... ... ... | 689 |
| **The Week's Work—** | |
| The Cost of Installing an Almoner—Pulmonary Tuberculosis at Isolation Hospitals—A Hospital Centenary—This Week's Drug Market ... ... ... ... ... ... ... ... ... | 691 |
| **Jottings** ... ... ... ... ... ... ... ... ... ... ... | 692 |
| **Parliament and Publications** ... ... ... ... ... | 692 |
| **Special Institutional Article—** | |
| Eminent Chairman Series, VI. The Hon. W. F. D. Smith ... | 693 |
| **Gifts and Bequests** ... ... ... ... ... ... ... ... | 696 |
| **Institutional Notes and News** ... ... ... ... ... ... | 697 |
| **Editor's Letter Box** ... ... ... ... ... ... ... ... | 698 |
| **Buildings Contemplated and in Progress** ... ... | 698 |
| **The Week's Novelties** ... ... ... ... ... ... ... | 698 |
| **New Appliances and Things Medical** ... ... ... ... | 698 |
| **Appointments** ... ... ... ... ... ... ... ... ... | 698 |

